# Increased aqueous humor levels of endothelin-1 in patients with open angle glaucoma

**DOI:** 10.1186/s12886-025-03861-y

**Published:** 2025-01-24

**Authors:** Adam Hedberg-Buenz, Erin A. Boese, Angela W. Nyunt, Nathan C. Sears, Andrew E. Pouw, Kai Wang, John H. Fingert

**Affiliations:** 1 Institute for Vision Research, University of Iowa, Iowa City, IA United States; 2 Department of Molecular Physiology and Biophysics, University of Iowa, Iowa City, IA United States; 3 Department of Ophthalmology and Visual Sciences, Carver College of Medicine, University of Iowa, Iowa City, IA United States; 4 Department of Biostatistics, College of Public Health, University of Iowa, Iowa City, IA United States; 5 3111B Medical Education and Research Facility, University of Iowa, 375 Newton Road, Iowa City, IA 52242 United States

**Keywords:** Endothelin-1, Aqueous humor, Primary open angle glaucoma, Exfoliation glaucoma, Normal tension glaucoma, ELISA, Intraocular pressure

## Abstract

**Background:**

Endothelin is a potent vasoconstrictor and contributes to the regulation of vascular perfusion. Aberrant endothelin-1 (ET-1) levels in aqueous humor have been reported across a variety of vascular diseases of the eye, including glaucoma. These findings suggest that dysregulation of ET-1 production may contribute to glaucoma pathophysiology. In this study, aqueous humor from patients undergoing ocular surgery was assayed for ET-1 abundance and related to the presence of glaucoma.

**Patients:**

Open angle glaucoma patients (*n*=62 total) from the ophthalmology clinics of the University of Iowa Hospitals and Clinics were enrolled in this study and organized into three distinct cohorts based on their diagnostic criteria, including those with primary open angle glaucoma (POAG, *n*=25 patients), normal tension glaucoma (NTG, *n*=17 patients), exfoliation glaucoma (XFG, *n*=8 patients), and normal controls (*n*=12 patients).

**Methods:**

Aqueous humor was collected intraoperatively from patients undergoing surgeries for glaucoma (including minimally invasive glaucoma surgeries, trabeculectomy, or glaucoma drainage device implantation) for samples in the glaucoma cohorts and cataract extraction for those in the control cohort. Aqueous humor was assayed by ELISA to measure and compare ET-1 abundance between the glaucoma cohorts and control cohort. ET-1 levels were also analyzed with linear regression to control for the covariates of age and sex.

**Results:**

ET-1 was significantly elevated in the aqueous humor of patients in the POAG (mean ± SD: 7.8 ± 5.1 pg/mL; p = 0.002) and NTG cohorts (6.1 ± 3.0 pg/mL; *p* = 0.030) compared to the control (4.0 ± 1.9 pg/mL). No significant difference in aqueous ET-1 was detected in the XFG cohort (6.2 ± 4.5 pg/mL; p = 0.230) compared to the control. Significantly higher ET-1 levels were detected in a merged grouping of all glaucoma cohorts (POAG, NTG, XFG) relative to controls (*p* = 0.021). Analysis of covariance indicated neither age nor sex was associated with ET-1 level (*p* = 0.60 and *p* = 0.27), respectively. Controlling for age and sex had minimal influence on the comparison of ET-1 levels in the POAG versus control cohort (*p* = 0.018) and nominal influence on the comparisons between the NTG (*p* = 0.089) or XFG cohort (*p* = 0.15) relative to the control.

**Conclusions:**

Elevated ET-1 in aqueous humor was associated with POAG and NTG compared to controls amongst cohorts of patients at the University of Iowa. These data suggest that dysregulation of vascular perfusion may have a role in the pathophysiology of POAG. The analyses of NTG and XFG samples were limited by the relatively small sample sizes.

**Supplementary Information:**

The online version contains supplementary material available at 10.1186/s12886-025-03861-y.

## Background

The glaucomas are a group of optic neuropathies that share key features of optic nerve damage (optic disc cupping) and visual field loss. Many key aspects of glaucoma pathophysiology are unknown. However, high intraocular pressure (IOP) is known to be a strong risk factor for glaucoma [1, 2] and causes dysfunction and damage to the optic nerve head at the level of the lamina cribrosa [3, 4]. Vascular factors have also been implicated in glaucoma pathophysiology. Nocturnal hypotension [5] and poor perfusion pressure [6–8] have been associated with primary open angle glaucoma (POAG).

Endothelin-1 (ET-1) is a powerful vasoconstrictor [9] that is secreted into the aqueous humor by the non-pigmented epithelium of the ciliary body [10]. High levels of ET-1 have been detected in the aqueous humor of patients with a range of vascular diseases, including diabetic retinopathy [11, 12] and branch or central retinal vein occlusion (BRVO or CRVO). Increased ET-1 levels in aqueous have also been detected in patients with a variety of different types of open angle glaucoma, including normal tension glaucoma (NTG), POAG, and exfoliation glaucoma (XFG) [13–20]. The observation of aberrant endothelin levels in aqueous humor of glaucoma patients has suggested that dysregulation of its production may contribute to glaucoma pathophysiology, perhaps by leading to vasoconstriction and compromised retinal and optic nerve perfusion [19, 21]. To our knowledge, few studies of ET-1 levels in aqueous humor have been conducted in glaucoma patients from the United States. Consequently, we investigated the aqueous humor levels of patients undergoing ocular surgery to determine if abnormally high ET-1 levels may be associated with the pathophysiology in our POAG, NTG, or XFG patients at the University of Iowa.

## Methods

### Patients

All research was conducted with the approval of the University of Iowa’s Institutional Review Board and adhered to the tenets of the Declaration of Helsinki. All patients provided informed written consent. Patient recruitment began on July 27, 2020 and ended on April 23, 2023. Patients and controls were consecutively invited to enroll in the study. All patients with glaucoma and normal control subjects were examined by board-certified ophthalmologists with fellowship training in glaucoma.

Patients were diagnosed with open angle glaucoma if they had open iridocorneal angles on gonioscopic exam and met the standard inclusion criteria of 1) stereotypical optic disc cupping and/or 2) glaucomatous visual field defects that we, [22–24] and others, have described [25]. *Exfoliation glaucoma (XFG)*. Patients diagnosed with open angle glaucoma, as outlined above, with additional signs of exfoliation syndrome (exfoliation material on the lens, exfoliation material in the trabecular meshwork, and moth-eaten pupillary margin) were diagnosed with exfoliation glaucoma. *Normal tension glaucoma (NTG)*. Patients with no signs of secondary glaucomas (i.e. pigment dispersion syndrome, exfoliation syndrome, steroid-induced glaucoma, or traumatic glaucoma) that had a maximum IOP of ≤ 21 mmHg were diagnosed with normal tension glaucoma. *Primary open angle glaucoma (POAG)*. Patients with no signs of secondary glaucomas (i.e. pigment dispersion syndrome, exfoliation syndrome, steroid-induced glaucoma, or traumatic glaucoma) that had a maximum IOP of > 21 mmHg were diagnosed with primary open angle glaucoma. *Control subjects*. All control subjects were found to have no glaucoma based on appearance of the optic disc and IOP of ≤ 21 mmHg.

### Aqueous humor collection

All aqueous humor samples were collected over a three-year period, beginning August 5, 2020 and ending on April 23, 2023. Aqueous humor samples were collected as the first intraocular step of surgery using a sterile canula, immediately placed on dry ice and transferred to a −70˚C freezer for safe and stable storage.

### Endothelin-1 ELISA

Aqueous humor samples were assayed using the ET-1 Quantikine ELISA kit (DET100; R&D Systems) on a 96 well microtiter plate and following the manufacturer’s protocol. ET-1 standards were processed concurrently with experimental samples. All samples (one per patient) from cases and controls were analyzed simultaneously after first thaw from storage and on the same plate in a single experiment. Samples from cases and controls were distributed randomly across the plate. A majority of samples (69%, 43 of the 62 total samples) were analyzed undiluted. A minority of samples (31%, 19 of 62), in which the volume collected was less than the minimum for the assay, were brought up to that minimum reaction volume using the kit’s calibrator diluent, and the final ET-1 quantification was scaled up (i.e. by multiplying the quantity of ET-1 detected in the well by the dilution factor used for achieving the minimum reaction volume in that same well) according to the dilution used. The absorbance OD450 nm (minus OD540 nm, for wavelength correction to account for the potential of optical interference due to imperfections in the plate and among others) for all reaction wells was measured using a Cytation 5 Cell Imaging Multimode plate reader (BioTek). Quantifications of ET-1 in aqueous humor were expressed in terms of concentration (pg/mL) and presented as mean concentration ± standard deviation for each of the three glaucoma cohorts and control cohort.

Aqueous humor samples were collected for this study from patients meeting the diagnostic criteria for glaucoma or control cohorts, as mentioned above in the “Patients” subsection of the methods. Patients in the POAG cohort were ranked by their maximum measured IOP and samples from the male and female patients with highest IOP were selected for inclusion in this study to facilitate comparisons with those in the NTG cohort (*n *= 25 total).

### Statistics

Demographic data were compared across each of the glaucoma cohorts and control cohort using a Fisher’s exact test for race and gender and a Mann-Whitney U-test test for age at enrollment. Comparisons amongst the three glaucoma cohorts were made using a Kruskall-Wallis test. Linear regression analysis was conducted with age and sex included as covariates. Ethnicity was not examined as a potential covariate since all subjects were non-Hispanic. For quantifications of ET-1 levels, a two-tailed Welch’s t-Test without correction for multiple comparisons was used to compare each of the three glaucoma cohorts to the control. Regression analysis and log transformation of the ET-1 data was used when samples from all three glaucoma cohorts (NTG, POAG, and XFG) were combined into one group and compared to the control cohort. Pearson’s correlation coefficients (R) were calculated to identify potential relationships between ET-1 concentration and either 1) the duration of storage of aqueous humor samples or 2) patient IOP.

## Results

### Demographics

Samples were collected from a total of (*n* = 62) patients, which included 25 with POAG, 17 with NTG, 8 with XFG, and 12 normal controls. Demographic data (age at enrollment, sex, race, and ethnicity) were collected from each of these cohorts (Table 1).

**Table 1:** Demographic features of study participants

Parameter	Cohorts				Statistical test	*p*-value
	Controls	POAG	NTG	XFG		
	(*n *= 12)	(*n *= 25)	(*n *= 17)	(*n *= 8)		
Age at enrollment (years)	67.3 ± 8.2	69.3 ± 9.1	72.9 ± 6.6	78.8 ± 3.2	Kruskall-Wallis test	*p *= 0.0075
Sex						
Female	11 (92%)	11 (44%)	16 (94%)	4 (50%)	Fisher's exact test	*p = *0.0006
Male	1 (8%)	14 (56%)	1 (6%)	4 (50%)		
Race						
White	12 (100%)	23 (92%)	17 (100%)	8 (100%)	Fisher's exact test	*p *> 0.99
Black	0 (0%)	1 (4%)	0 (0%)	0 (0%)		
Asian	0 (0%)	1 (4%)	0 (0%)	0 (0%)		
Ethnicity						
non-Hispanic	12 (100%)	25 (100%)	17 (100%)	8 (100%)	Not tested.	
Hispanic	0 (0%)	0 (0%)	0 (0%)	0 (0%)		

Demographic data was compared using a series of statistical tests. Amongst all four cohorts (control, POAG, NTG, and XFG), there was a statistically significant difference in age of enrollment (*p* = 0.0075; Kruskall-Wallis test). Pairwise comparisons revealed no statistical difference in the age at enrollment for the POAG (*p* = 0.62) and NTG cohorts (*p* = 0.063) relative to the controls, however the XFG cohort (78.8 ± 3.2 years) was significantly older than controls (67.3 ± 8.2 years; *p* = 0.0019; Mann-Whitney). With respect to sex, there was a statistically significant difference in the proportion of sexes across the four cohorts (*p* = 0.0006; Fisher’s exact test). Pairwise comparisons showed no differences in the NTG (*p* > 0.05) and XFG cohorts (*p* > 0.05) relative to controls, however, there was a significantly lower proportion of females in the POAG cohort (44%) relative to the controls (92%; *p* = 0.011; Fisher’s exact test). Conversely, there were no significant differences in the proportions of race between the four cohorts (*p* > 0.99; Fisher’s exact test). Likewise, no significant difference in the proportions of ethnicity were detected across the four cohorts, since all participants were of the same ethnic group (non-Hispanic). These analyses identified age at diagnosis and sex as demographic parameters that should be analyzed as potential covariates in our proposed studies of ET-1 levels in the aqueous samples from the control, POAG, NTG, and XFG cohorts. Finally, patients in each of the three glaucoma cohorts (POAG, NTG, and XFG), had similar usage of topical glaucoma medications at the time of enrollment, including prostaglandin analogs, beta-blockers, alpha-agonists, rho-kinase inhibitors, cholinergics (S. Table 1).

### Endothelin-1 in aqueous humor samples

**Fig. 1:**
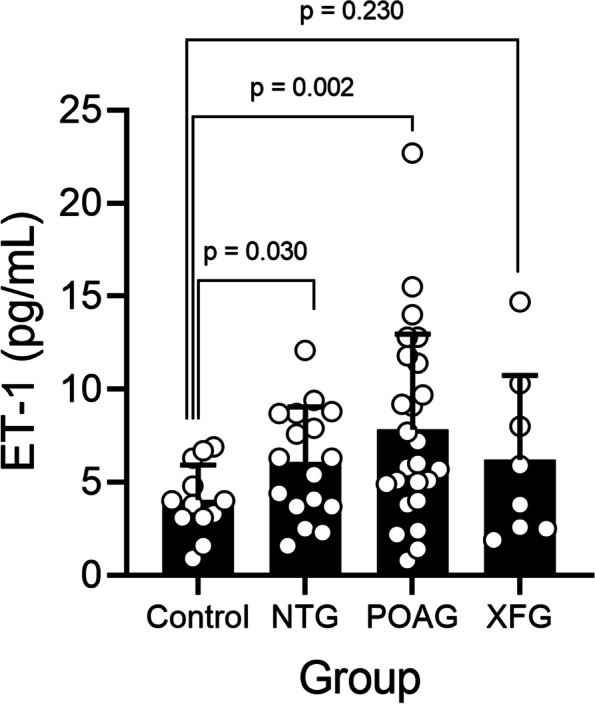
Endothelin-1 is elevated in aqueous humor in the normal tension and primary open angle glaucoma cohorts. Graph showing measurements of endothelin-1 (ET-1) concentration in aqueous humor from patients across three glaucoma cohorts, including normal tension glaucoma (NTG), primary open angle glaucoma (POAG), and exfoliative glaucoma (XFG) and a normal control cohort. Statistically higher concentrations of ET-1 were detected in aqueous humor samples from POAG (*n *= 25, *p* = 0.002) and NTG (*n *= 17, *p* = 0.030) compared to controls (*n *= 12). Despite a trend for higher concentrations of ET-1 in aqueous humor from patients in the XFG cohorts (*n *= 8, *p* = 0.15) relative to controls, this did not reach statistical significance. In this graph, each bar represents mean ET-1 concentration ± standard deviation, each dot represents data from one sample per patient within each cohort, and statistical analysis with inset *p*-values (calculated using a two-tailed Welch’s t-Test without correction for multiple comparisons).

The levels of ET-1 in aqueous humor (expressed as mean concentration ± standard deviation) were measured using a commercial ELISA assay for patients within each glaucoma cohort and compared with those in the control cohort (Fig. 1). The concentration of ET-1 was significantly higher in the aqueous humor from patients in the POAG (7.8 ± 5.1 pg/mL; *p* = 0.002) and NTG cohorts (6.1 ± 3.0 pg/mL; *p* = 0.030) compared to the control (4.0 ±1.9 pg/mL; two-tailed Welch’s t-Test). Despite a trend for higher ET-1 concentrations in the aqueous humor from patients in the XFG (6.2 ± 4.5 pg/mL; *p* = 0.230) compared to the control cohort (4.0 ±1.9 pg/mL; two-tailed Welch’s t-Test), this difference did not reach statistical significance.

Aqueous humor ET-1 concentrations were also assessed using linear regression analysis while controlling for the sex and age at enrollment of patients. Measurements of ET-1 were first log-transformed to reduce asymmetry in the distribution and then analyzed with regression (S. Figure 1). Notably, neither age at enrollment (*p* = 0.60) nor sex (*p* = 0.27) of patients were associated with log (ET-1) in this analysis. After controlling for sex and age at enrollment, ET-1 levels in the POAG cohort were statistically higher than in the control cohort (*p* = 0.018). However, no statistical differences were detected between the NTG (*p* = 0.089) or XFG cohort (*p* = 0.15), relative to the control. Finally, patients in all three glaucoma groups (POAG, NTG, and XFG) were combined into a single glaucoma cohort and compared to controls in an analysis using linear regression while controlling for sex and age at enrollment. Significantly higher ET-1 levels were detected in the aqueous humor of this combined glaucoma cohort relative to controls (*p* = 0.021).

**Fig. 2:**
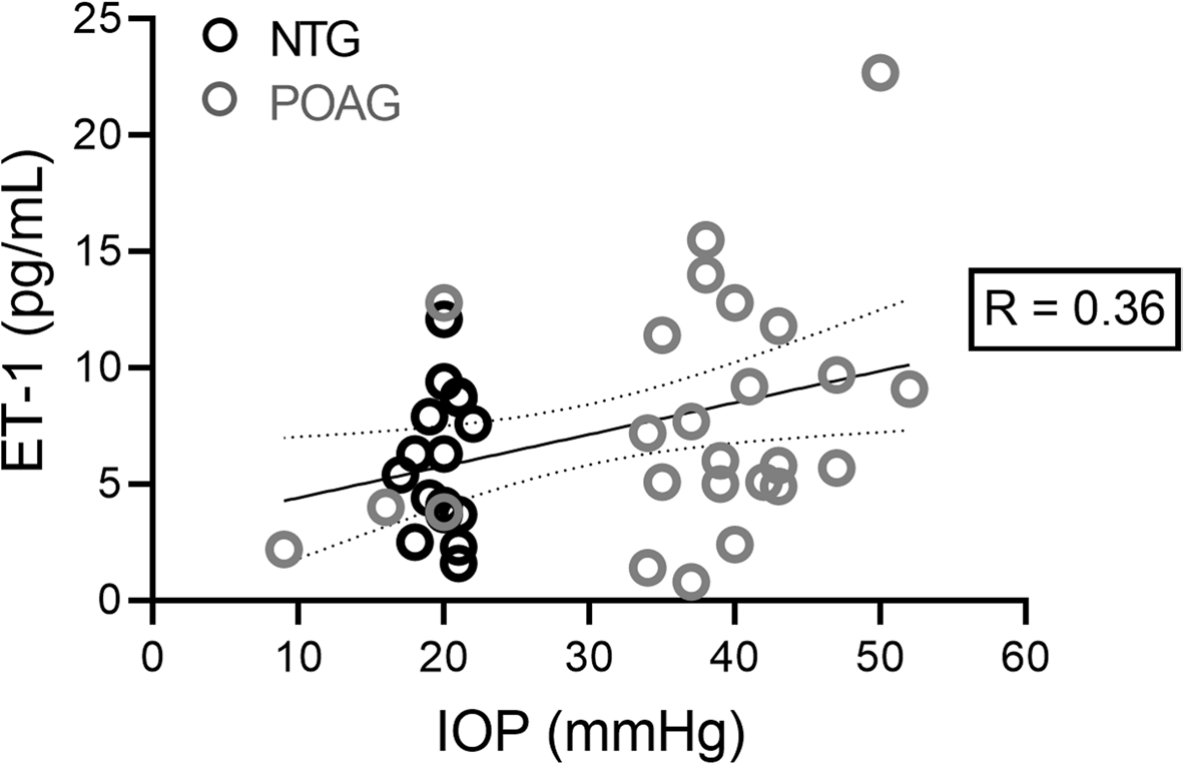
Relationship between endothelin-1 levels in aqueous humor and intraocular pressure among patients with normotensive or hypertensive open angle glaucoma. Graph plotting measurements of endothelin-1 (ET-1) concentration against the maximum intraocular pressure (IOP) measurement for each patient within the indicated cohort, which includes normal tension glaucoma (NTG; black dots) and primary open angle glaucoma (POAG; grey dots). Aqueous humor ET-1 concentration correlated positively (R = 0.36) with IOP. In this graph, the solid and dotted lines represent the line of regression and 95% confidence interval, respectively, inset Pearson’s correlation coefficient (R), and each dot represents data from one sample from one patient

Akin to quality control testing, we investigated the potential effects that duration of storage of aqueous humor samples had on ET-1 levels to address the possibility of molecule degradation or evaporative volume loss that may have occurred over the three-year period in which samples were collected and stored at −70˚C. No significant correlation between storage time and ET-1 concentration was detected within any of the study cohorts, and in all cohorts, the R ≤ 0.18 and slope of the regression lines were not significantly different from zero. We also assessed the potential for bias related to the dilution of a minority of aqueous samples before conducting ELISA experiments. ET-1 levels were compared between diluted and undiluted aqueous humor samples. No differences were detected between the mean level of ET-1 in each group, suggesting that sample dilution was not likely to bias the measurement (data not shown). In comparisons between the NTG and POAG cohorts, aqueous humor and IOP data for all patients were combined to relate ET-1 concentration with the maximum IOP measured for each patient. In this analysis, ET-1 concentration correlated positively with maximum IOP (R = 0.36) amongst patients in the NTG and POAG cohorts (Fig. 2).

## Discussion

Since it’s discovery in 1988 [9], ET-1 has long been implicated in glaucoma pathogenesis via its potent vasoconstriction activity [26–30]. ET-1 may compromise perfusion of retinal ganglion cells and the optic nerve by promoting vasoconstriction. Several reports of higher serum ET-1 levels in patients with POAG (1.1 to 2.1-fold higher than control) or NTG (1.1 to 3.3-fold higher than control) [31] support this vascular hypothesis for glaucoma pathogenesis. We report statistically higher levels of ET-1 in aqueous humor samples from patients with POAG (2.0-fold higher, *p* = 0.002) and NTG (1.5-fold increase, *p* = 0.030) when compared to normal controls from Iowa. Although aqueous ET-1 levels were higher in the XFG cohort (1.6-fold higher, *p* = 0.23) relative to controls, this difference was below the threshold for statistical significance, which may be due to the smaller sample size of this cohort. Our analyses suggested that age and sex are not associated with aqueous humor levels of ET-1. Nonetheless, we reanalyzed ET-1 levels with age and sex as covariates using linear regression. Aqueous humor levels of ET-1 were statistically higher in the POAG cohort than the control cohort with age and sex as covariates (*p* = 0.018). However, the higher ET-1 levels detected in NTG and XFG cohorts did not meet threshold for statistical significance, likely due in part to smaller sample sizes. The discovery of higher ET-1 levels in the aqueous humor of our cohorts of POAG, NTG, and XFG patients from Iowa is validated by similar results in prior studies. In 1997, Tezel and colleagues detected statistically higher endothelin in aqueous humor from POAG patients [27] as did Noske and coworkers [32]. Similar to our findings, Tezel et. al. found that age and sex were not associated with aqueous ET-1. Noske et. al. did not include age and sex as covariates in their analyses. Several subsequent studies have also confirmed the presence of elevated endothelin in the aqueous humor of patients with POAG [17, 19, 33] and animal models of glaucoma [34]. Prior investigations of patients with exfoliation syndrome and XFG also detected higher aqueous humor levels of endothelin when compared with controls [13–16, 35]. A recent systemic meta-analysis found that elevated ET-1 in aqueous humor is significantly associated with POAG and XFG [36]. Thus, our report provides additional support for an association between elevated endothelin levels in aqueous humor and POAG, NTG, and XFG.

There are some limitations to this study. First, our study cohorts are relatively small. This may impede the detection of an association between aqueous ET-1 level and glaucoma, and increase susceptibility to biased results, especially for the smallest cohort of XFG patients. Second, our cohorts are representative of the homogeneous population in Iowa, which is primarily non-Hispanic white, and may not be generalizable to other populations. Third, we preferentially enrolled POAG patients with higher IOP to facilitate comparisons with the NTG cohort in our study, as described in the Methods section. Consequently, there was bias for selection of samples from patients with greater maximum IOP and potentially higher aqueous ET-1 levels, since aqueous ET-1 levels have been shown to correlate with IOP [13]. Thus, our findings may be more representative of POAG patients with relatively higher IOP. Fourth, although our glaucoma and control cohorts were well-matched for race and ethnicity, there were differences in the age at enrollment and sex of patients between cohorts (Table 1). For example, it was determined that patients in the XFG cohort were older at the time of enrollment than in controls. Though this finding may be explained in part by the fact that a late onset of disease is a known feature of XFG, it nonetheless makes age-matching for controls and avoiding bias for age more challenging. As another example, it was determined that the proportion of females in the NTG and control cohorts was greater than in the POAG and XFG cohorts. However, these potential biases have been addressed. Our analyses showed that age of enrollment and sex were not associated with aqueous ET-1 concentration in our patients (S. Figure 1), suggesting that the results were not confounded by these potential biases. Fifth, the glaucoma patients in this trial were taking numerous topical glaucoma medications, while the control subjects were not. It is unknown whether these medications might have an influence on ET-1 levels. Similarly, systemic medications taken by patients and controls may also have influenced ET-1 levels and biased our results. Despite these limitations, our study provides additional data linking ET-1 with glaucoma pathogenesis in patients from the United States, which is supported by previous reports of this same linkage in other disparate patient populations from Germany, Greece, Mexico, and Iran [15–17, 19].

Mechanisms by which ET-1 may promote retinal ganglion cell death and glaucoma remain unclear. In addition to ischemic damage, ET-1 may also directly promote retinal ganglion cell damage by stimulating programmed cell death [21, 37]. ET-1 may also influence IOP and risk for glaucoma. An association has been reported between aqueous humor levels of ET-1 and IOP [13]. Moreover, endothelin has been shown to influence facility of outflow in bovine, [38] rabbit [39] and non-human primate eyes [28]. Thus, in addition to inducing vasoconstriction and reducing perfusion, ET-1 may also contribute to glaucoma by regulating aqueous fluid dynamics and IOP. Research to investigate mechanisms by which endothelin influences glaucoma pathophysiology has great potential to clarify the causes of glaucoma, identify new therapeutic targets, and novel treatment strategies.

## Conclusions

In this study, the abundance of ET-1 in aqueous humor from cohorts of patients with distinct types of open angle glaucoma were measured and compared with that from control patients at the University of Iowa Hospitals and Clinics. ET-1 concentration was significantly higher in the aqueous humor from patients with POAG and NTG, compared to controls. Although there was a trend for higher aqueous ET-1 in patients with XFG relative to controls, this difference was not significant. In an analysis that included patients from all three glaucoma groups combined into a single cohort, ET-1 was significantly higher in the aqueous humor from patients in the glaucoma cohort compared to the control, suggesting that endothelin levels may have an important role in glaucoma pathogenesis.

## Supplementary Information

Supplementary Material 1: Supplementary Table 1. Glaucoma medications of study subjects

Supplementary Material 2: Supplementary Figure 1. Log-transformation of endothelin-1 measurements by the sex and age of enrollment of subjects in each of the cohorts

Supplementary Material 3.

## Data Availability

Data is provided within the manuscript and supplementary information files.

## Abbreviations

ET-1: Endothelin-1
POAG: Primary open angle glaucoma
NTG: Normal tension glaucoma
XFG: Exfoliation glaucoma
IOP: Intraocular pressure
R: ‘Pearson’s correlation coefficient
