# Supplementary material for: Increased aqueous humor levels of endothelin-1 in patients with open angle glaucoma
Source: BMC Ophthalmol. 2025 Jan 24;25:46. doi: 10.1186/s12886-025-03861-y (PMC11760677; doi:10.1186/s12886-025-03861-y)
Supplement: Supplementary file 1 — Supplementary Material 1: Supplementary Table 1. Glaucoma medications of study subjects [file 12886_2025_3861_MOESM1_ESM.pdf]

	Prostaglandin analogs	Beta-blockers	Carbonic anhydrase inhibitors	Alpha agonists	Rho kinase inhibitors	Latanoprostene bunod	Cholinergics	Methazolamide
POAG (n = 25)	20 (80.0%)	21 (84.0%)	12 (48.0%)	17 (68.0%)	4 (16.0%)	1 (4.0%)	1 (4.0%)	1 (4.0%)
NTG (n=17)	12 (70.6%)	13 (76.5%)	8 (47.1%)	6 (35.3%)	5 (29.4%)	0	0	0
XFG (n=8)	7 (87.5%)	8 (100%)	3 (37.5%)	2 (25%)	0	0	0	0

Supplementary Table 1. Glaucoma medications of study subjects.
