# Supplementary figures and images for: Increased aqueous humor levels of endothelin-1 in patients with open angle glaucoma
Source: BMC Ophthalmol. 2025 Jan 24;25:46. doi: 10.1186/s12886-025-03861-y (PMC11760677; doi:10.1186/s12886-025-03861-y)

Supplementary Figure 1. Log-transform of endothelin-1 measurements.

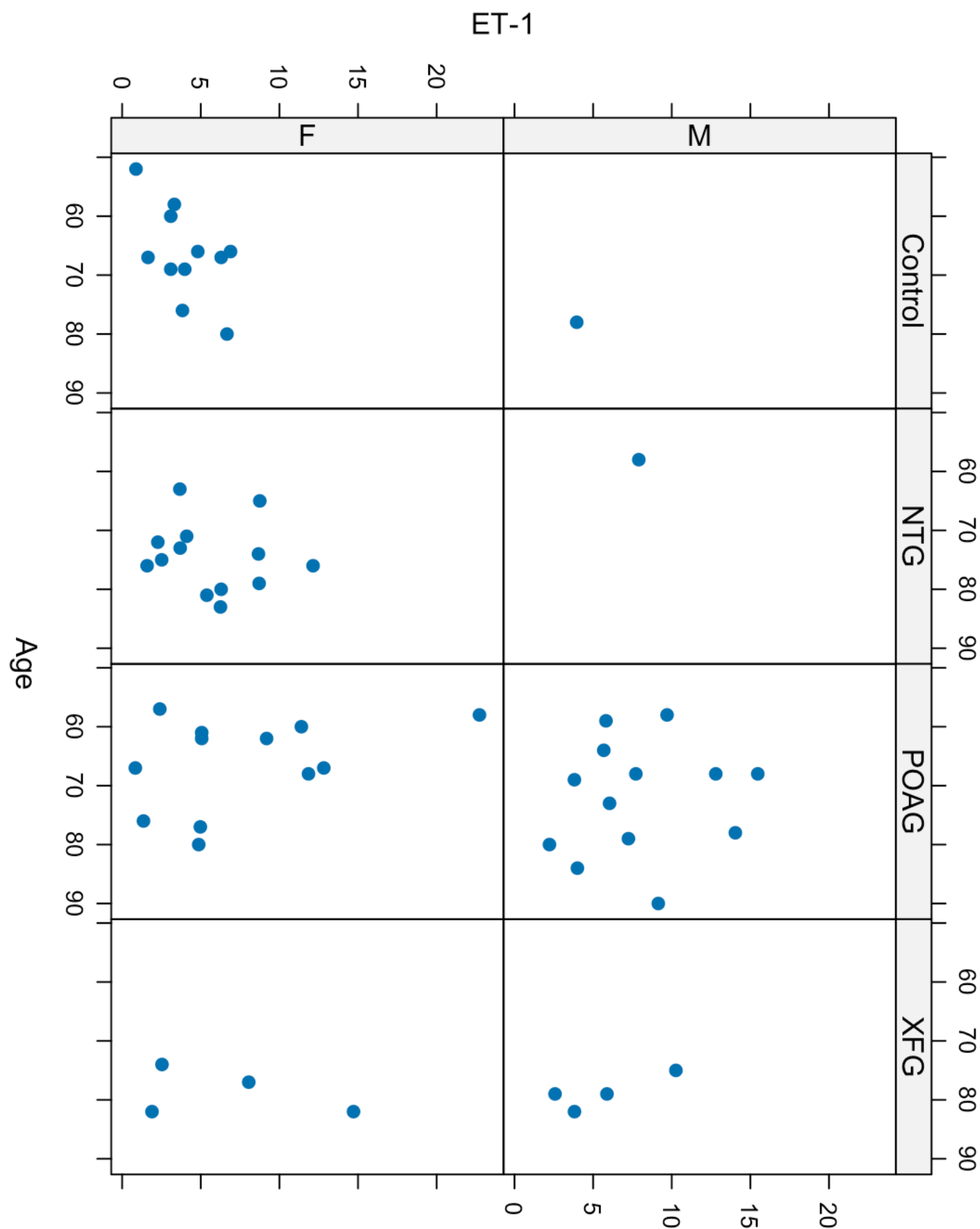

Supplement: Supplementary file 2 — Supplementary Material 2: Supplementary Figure 1. Log-transformation of endothelin-1 measurements by the sex and age of enrollment of subjects in each of the cohorts [file 12886_2025_3861_MOESM2_ESM.pdf]
